# APOE2: protective mechanism and therapeutic implications for Alzheimer’s disease

**DOI:** 10.1186/s13024-020-00413-4

**Published:** 2020-11-04

**Authors:** Zonghua Li, Francis Shue, Na Zhao, Mitsuru Shinohara, Guojun Bu

**Affiliations:** 1 Department of Neuroscience, Mayo Clinic, Jacksonville, FL USA; 2 Neuroscience Graduate Program, Mayo Clinic, Jacksonville, FL USA; 3 Department of Aging Neurobiology, National Center for Geriatrics and Gerontology, 7-430 Morioka, Obu, Aichi 474-8511 Japan

**Keywords:** Apolipoprotein E2, Alzheimer’s disease, Amyloid-β, Cerebrovascular disease, Lipid metabolism, Longevity, Neuroinflammation, Neurofibrially tangles, TDP-43, α-Synuclein

## Abstract

Investigations of apolipoprotein E (*APOE*) gene, the major genetic risk modifier for Alzheimer’s disease (AD), have yielded significant insights into the pathogenic mechanism. Among the three common coding variants, APOE*ε4 increases, whereas APOE*ε2 decreases the risk of late-onset AD compared with APOE*ε3. Despite increased understanding of the detrimental effect of APOE*ε4, it remains unclear how APOE*ε2 confers protection against AD. Accumulating evidence suggests that APOE*ε2 protects against AD through both amyloid-β (Aβ)-dependent and independent mechanisms. In addition, APOE*ε2 has been identified as a longevity gene, suggesting a systemic effect of APOE*ε2 on the aging process. However, APOE*ε2 is not entirely benign; APOE*ε2 carriers exhibit increased risk of certain cerebrovascular diseases and neurological disorders. Here, we review evidence from both human and animal studies demonstrating the protective effect of APOE*ε2 against AD and propose a working model depicting potential underlying mechanisms. Finally, we discuss potential therapeutic strategies designed to leverage the protective effect of *APOE2* to treat AD.

## Background

Apolipoprotein E (*APOE*), as an apolipoprotein mediating lipid metabolism in circulation and the brain, is the strongest genetic risk modifier of late-onset Alzheimer’s disease (LOAD, referred to as AD in this review) [1–4]. Among the three common coding variants of APOE, APOE*ε4 increases, whereas APOE*ε2 decreases, the risk of AD compared with the most common APOE*ε3 allele [5, 6]. The mechanism underlying the protective effect of APOE*ε2 against AD remains unclear. Human studies show that APOE*ε2 is associated with reduced Aβ deposition in the brains of non-demented aged individuals and AD patients [7–11], suggesting that APOE*ε2 reduces AD risk at least partially through Aβ-dependent pathways. APOE*ε2 may also protect against AD through Aβ-independent pathways. Supporting this, APOE*ε2/2 and APOE*ε2/3 individuals (referred to as APOE*ε2 carriers in this review) are more likely to be cognitively intact compared with APOE*ε3/3 homozygotes among individuals with minimal Aβ pathology [12]*.* In addition, studies show that APOE*ε2 protects against cognitive impairment in individuals over 90 years of age who have high levels of Aβ in the brain [13, 14]. In vitro and in vivo studies suggest multiple potential pathways through which APOE2 confers protection independently of Aβ pathology. These pathways likely involve the neuroprotective effect of APOE2 and the regulatory roles of APOE2 in lipid metabolism and synaptic functions [15–18].

Although APOE*ε2 has also been associated with longevity [19–23], which might be independent of its protective role against AD [24, 25], it is not entirely benign. APOE*ε2 is associated with an increased risk of cerebral amyloid angiopathy (CAA) which often co-exists with AD pathology and is a major cause of hemorrhagic stroke [26, 27]. APOE*ε2 is also associated with increased risk of certain neurological disorders such as post-traumatic stress disorder (PTSD) [28], age-related macular degeneration (AMD) [29], supranuclear palsy (PSP), and argyrophilic grain disease (AGD) [30, 31]. In this review, we summarize recent progress in APOE*ε2 research and propose a hypothetical working model depicting the protective effect of APOE*ε2 against AD. We also discuss potential therapeutic strategies for AD inspired by APOE*ε2-related protective mechanisms.

## Main text

### Biology of APOE

#### Human APOE

**Fig. 1:**
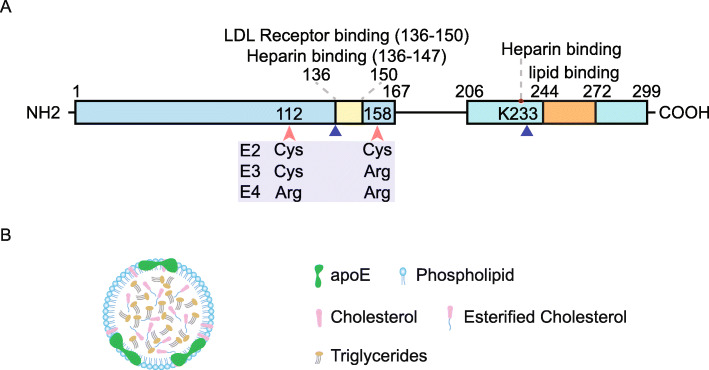
Human APOE. **a** Human APOE is an O-linked glycoprotein consisting of 299 amino acids. The N-terminal domain (residues 1-167) and the C-terminal domain (residues 206-299) are linked by a flexible hinge region. The receptor binding site (residues 136-150) on the N-terminus overlaps with the heparin binding region (residues 136-147). A second heparin-binding site on the C-terminal domain adjacent to the lipid binding site (residue 244-272) requires K233. Amino acid substitutions at position 112 and 158 result in the three major isoforms: APOE2 (Cys112; Cys158), APOE3 (Cys112; Arg158) and APOE4 (Arg158; Arg158). APOE has other less common isoforms; APOE (V236E) and APOE3 Christchurch (R136S) (blue triangles) are two examples that have also been suggested to protect against AD. **b** Lipidated APOE-containing lipoprotein particles contain phospholipids and unesterified cholesterol in the shell, and esterified cholesterol and triglycerides form the core. APOE molecules are partially embedded in the phospholipid layer of the particles

Human APOE is a 34-kDa glycoprotein consisting of 299 amino acids [32], encoded by the *APOE* gene located on chromosome 19q13.32 [33]. The three allelic variants, namely, APOE*ε2, APOE*ε3, and APOE*ε4, encode three isoforms that differ from each other at two amino acid positions 112 and 158: APOE2 (Cys^112^; Cys^158^), APOE3 (Cys^112^; Arg^158^), and APOE4 (Arg^112^; Arg^158^) [32, 34]. Structurally, APOE has two independently-folded domains referred to as the N-terminal domain and the C-terminal domain [35, 36] (Fig. 1a). These two domains are linked by a flexible loop region that is thrombolytically cleavable [37, 38]. The N-terminal domain contains the receptor-binding site (residues 136-150) [39], whereas the C-terminal domain contains the lipid-binding region (residues 244-272) [4, 40]. Additionally, residues 136-147 in the N-terminal domain and the basic residue Lys^233^ in the C-terminal domain are required for APOE binding to heparin/heparan sulfate polysaccharide chains of HSPG, another important receptor of APOE [41, 42].

In humans, peripheral and central nervous system (CNS) APOE do not cross the blood-brain barrier (BBB), thus forming two independent APOE pools with no APOE-containing lipoprotein exchange [43]. In the periphery, APOE is produced primarily by liver hepatocytes [44], while in the CNS, the majority of APOE derives from astrocytes, microglia, vascular mural cells, and the choroid plexus [45, 46]. Stressed neurons also produce APOE, albeit to a much lesser extent [45, 47]. APOE levels in human plasma follow the *APOE* genotype rank order of APOE*ε2/2 > APOE*ε2/3 > APOE*ε3/3 (or APOE*ε2/4) > APOE*ε3/4 > APOE*ε4/4 [48–51]. In contrast, the impact of *APOE* genotype on CSF APOE levels varies across studies with different quantification methods. While enzyme-linked immunosorbent assay (ELISA)-based measurements show a similar *APOE* genotype effect to that in plasma [48], mass-spectrometric assays find no such effect [52, 53]. Similar to results from human plasma, cortical APOE levels measured by Western blot and ELISA are highest in APOE*ε2 carriers and lowest in APOE*ε4 carriers [54]. This is consistent with observations from APOE-targeted replacement (APOE-TR) mice in which the murine *Apoe* gene locus is replaced with human *APOE* alleles [55], showing that APOE2-TR mice have higher levels of APOE in the interstitial fluid (ISF) and brain lysate than APOE3-TR mice, followed by APOE4-TR mice [56–58].

#### APOE receptors

APOE functions through binding to cell surface receptors, including low-density lipoprotein receptor (LDLR), very low-density lipoprotein receptor (VLDLR), LDLR-related protein 1 (LRP1), APOE receptor 2 (APOER2, also known as LRP8), and heparan sulfate proteoglycans (HSPGs) [59–61]. In addition, recent studies show the triggering receptor expressed on myeloid cells 2 (TREM2), which is specifically expressed by microglia in the brain, is a receptor for APOE [62–64]. The interaction between APOE and receptors shows isoform-specificity and is affected by APOE lipidation status (Table 1), which is best exemplified by LDLR that recognizes only lipidated APOE [67–69], and shows much weaker binding to APOE2 relative to APOE3 and APOE4 [65, 66].

**Table 1:** APOE receptors

APOE receptors	Isoform-specific binding	APOE lipidation required for receptor binding?	APOE binding related functions
LDLR	Lipidated APOE: APOE2 < <APOE3 = APOE4 [65, 66]	Yes [67–69]	Mediates lipoprotein and Aβ clearance [4, 70]
LRP1	Lipidated APOE: APOE2 < APOE3 = APOE4 [71]; Non-lipidated APOE: APOE3 binds immobilized LRP1 recombinant cluster IV with a higher affinity than APOE4 [72]	Likely not required although one study suggests otherwise [67, 72, 73]	Mediates lipoprotein and Aβ clearance [70, 74]; signal transduction [15–17]; neurotrophic effect [16, 75–80].
VLDLR	Non-lipidated APOE: APOE2 = APOE3 = APOE4 [67]	No [67]	Mediates lipoprotein and Aβ clearance [70, 81], as well as reelin signaling [82–84].
APOER2/LRP8	Non-lipidated APOE: APOE2 < <APOE3 = APOE4 [85]	No [85]	Mediates reelin signaling [82–84]; regulates intracellular trafficking of synaptic receptors [18].
HSPG	Non-lipidated APOE: APOE2 < APOE3 < APOE4 [86]	No [41, 86]	Mediates lipoprotein and Aβ clearance [4, 70]
TREM2	Both lipidated and non-lipidated APOE: APOE2 = APOE3 = APOE4 [62–64]	No [62–64]	Mediates microglial phagocytosis of Aβ and damaged neurons [64, 87, 88]; Maintains neurodegenerative phenotype (MGnD) of disease-associated microglia (DAM) [89, 90].

APOE binding to receptors either triggers the uptake of APOE or activates downstream singling cascades involving primarily mitogen-activated protein (MAP) kinases [15–17, 61]. The APOE receptor-mediated ligand uptake represents the major mechanism of lipoprotein clearance in the periphery and lipid transport in the CNS [70, 91]. However, the physiological role of APOE-triggered signaling pathways is less clear. In vitro studies show that APOE, regardless of the lipidation status, triggers diverse signaling pathways in neurons, likely through LRP1, to support versatile functions such as neuronal protection and synaptogenesis [15–17]. The functional significance of the interaction between APOE and TREM2 remains to be elucidated, although evidence suggests a role in microglia-mediated clearance of Aβ and damaged neurons [64, 87].

### Biological functions of APOE

#### APOE and lipid metabolism

In the periphery, APOE plays a major role in mediating the clearance of triglyceride-rich lipoproteins (chylomicrons, VLDL, and their remnants) by interacting with hepatic APOE receptors [70]. Individuals of different *APOE* genotypes differ in their plasma lipid profiles. Compared with APOE*ε3/3 homozygotes, APOE*ε3/4 and APOE*ε4/4 individuals (referred to as APOE*ε4 carriers in this review) exhibit higher levels of total cholesterol, LDL, and triglycerides (TGs), and lower levels of HDL, whereas APOE*ε2 carriers have lower levels of total cholesterol and LDL, and higher levels of HDL and TGs in the plasma [92, 93]. The *APOE* genotype-specific plasma lipid profile is a combinatory result of multiple factors [70, 94]. For example, while impaired binding of APOE2 to LDLR is causally linked to type III hyperlipoproteinemia, characterized by the accumulation of remnants of TG-rich lipoproteins [65, 66, 94, 95], hyperlipidemia is only observed in 5-10% of APOE*ε2/2 homozygotes [94]. The majority of APOE*ε2 carriers have normal or, paradoxically, hypolipemic profile, which is thought to be partially caused by the lower efficiency of lipolytic conversion of APOE2-containing VLDL and IDL to HDL [96–98]. Notably, the lipid profile of APOE2-TR mice resembles the small portion of human APOE*ε2 homozygotes who develop hyperlipidemia [99], raising cautions when interpreting results from studies using APOE2-TR mice.

In the CNS, APOE is the major apolipoprotein that transports lipids [91]. CNS APOE is lipidated by cell surface ATP-binding cassette transporters ABCA1 or ABCG1 [100–103]. Lipidated APOE forms HDL-like particles in size and density containing free cholesterol and phospholipids [104–106]. Brain-specific deficiency of *Abca1* in mice results in impairments in motor activity and sensorimotor functions, and changes in synaptic structures [107], suggesting a crucial role of APOE-mediated lipid metabolism in the CNS. However, no substantial difference in the brain lipidomics profile has been identified between APOE2-TR, APOE3-TR, and APOE4-TR mice at young and middle-age [108], although aged APOE2-TR mice have lower cortical cholesterol levels than APOE3-TR and APOR4-TR mice [12]. In human AD brains, APOE*ε2 carriers and APOE*ε3/3 homozygotes have similar lipidomics profiles, whereas APOE*ε4 carriers have a significant reduction in ten major lipid classes, including phosphatidylethanolamine, phosphatidic acid, and mitochondrial membrane bilayer-forming phospholipids [109]. Future studies elucidating the role of APOE isoforms in cell type-specific lipid metabolism may aid our understanding of the mechanisms underlying APOE-associated AD risks.

#### Neurotrophic effect of APOE

The neurotrophic effect of APOE has been well-documented. However, questions remain regarding isoform-specific effects. APOE3, regardless of the lipidation status, promotes neurite outgrowth through a mechanism depending on LRP1, whereas APOE4 has no effect or inhibitory effect [75–80]. In addition, APOE3-containing HDL lipoprotein particles protect neurons from apoptosis induced by nutrient depletion at a higher efficiency than APOE4-containing particles, which requires LRP1 as well [16]. APOE also promotes synaptogenesis through mediating cholesterol transport from astrocytes to neurons [110]; however, it is unclear whether the effect is APOE isoform-specific. The neurotrophic effect of APOE2 relative to those of APOE3 and APOE4 has been less studied. Although APOE2-TR mice displayed longer dendritic spines and increased apical dendritic arborization in the cortex at one month of age compared with APOE3-TR mice, the differences have not been observed in older animals [111]. Moreover, there is no difference in dendritic spine density in the hippocampus of APOE2-TR, APOE3-TR, and APOE4-TR mice at different ages [111].

#### APOE and synaptic functions

Synaptic dysfunction is one of the earliest pathological changes in AD [112, 113]. In vitro data suggest a regulatory role of APOE in synaptic functions. Astrocyte-derived APOE4, but not APOE2 or APOE3, reduces the levels of postsynaptic APOER2, N-methyl-D-aspartate receptor (NMDAR), and α-amino-3-hydroxy-5-methyl-4-isoxazolepropionic acid receptor (AMPAR) in cultured neurons by sequestering the receptors in the intracellular compartment [18]. Additionally, lipidated APOE2 enhances, whereas APOE4 suppresses glutamate-induced calcium influx through NMDAR in the presence of Reelin [18]. Lipidated APOE2 also enhances the elimination of synapses by astrocytes more than APOE3 and APOE4 in culture, indicating that APOE2 may protect synaptic functions by reducing senescent synapses and the accumulation of neural debris [114].

In vivo, young adult APOE2-TR, APOE3-TR, and APOE4-TR mice have similar levels of postsynaptic density protein 95 (PSD-95) in the cortex and hippocampus [12]. However, electrophysiological studies show comparable or lower LTP amplitudes in APOE2-TR mice compared with APOE3-TR mice [115, 116]. The absence of an APOE isoform effect on synaptic functions in young APOE-TR mice is not surprising given comparable cognitive performance between APOE2-TR, APOE3-TR, and APOE4-TR mice, and between humans of different APOE genotypes at young ages (< 60 years old) [12, 117, 118]. Since the protective effect of APOE2 against cognitive decline is most prominent in the elderly [12, 119, 120], one would assume a better synaptic function in aged APOE2-TR mice compared with APOE3-TR and APOE4-TR mice of the similar age. Indeed, one study show that aged APOE4-TR mice display poorer spatial memory acquisition, whereas APOE2-TR mice exhibit better spatial memory retention than APOE3-TR mice [12].

#### APOE and innate immunity

Innate immunity plays a crucial role in AD pathogenesis [121–125]. The involvement of APOE in AD-associated immune response is evident in recent transcriptomics studies [89, 90, 126–128]. In amyloid mouse models, APOE upregulation is a major molecular signature of the subtype of microglia known as disease-associated microglia (DAM) [89, 90, 126]. The acquisition of the neurodegenerative phenotype (MGnD) of DAM is driven by Aβ plaques via a TREM2-dependent pathway [89, 90]. *Trem2* knockout abolishes Aβ-driven upregulation of *Apoe* and reduces plaque-associated APOE protein in amyloid mouse models [89, 129]. Consistent with these findings, microglial APOE is also upregulated in pathologically-confirmed human AD brains [126, 127].

APOE likely modulates microglial function in an isoform-dependent manner through TREM2-mediated pathways [130]. However, it remains unclear how APOE isoforms differentially regulate the immune response, in particular in AD pathogenesis. Evidence from studies of lipopolysaccharide (LPS)-induced immune reactivity shows a greater response associated with APOE*ε4 [131–134]. However, there are conflicting results regarding the regulatory role of APOE*ε2 in innate immunity. Although one study showed that microglial culture derived from APOE2-TR mice display reduced immune response upon LPS treatment than that derived from APOE3-TR mice [134], others found no such difference [131]. Moreover, APOE2-TR and APOE3-TR mice show comparable cytokine release and glial activation after intracerebroventricular LPS injection [135]. As LPS treatment induces acute immune responses, which does not capture the AD-related conditions, future studies on APOE isoform-specific role in innate immunity should be carried out with AD mouse models bearing amyloid and/or tau pathology.

#### APOE and blood-brain barrier integrity

BBB breakdown is present in multiple neurodegenerative diseases, including AD [136]. Animal studies show that APOE*ε4 correlates with decreased BBB integrity [137] and slower BBB repair after brain injury [138], which is consistent with the observation in humans that aged APOE*ε4 carriers have increased BBB permeability compared with APOE*ε3 homozygotes, irrespective of the cognitive status [139]. Moreover, the association between APOE*ε4 and BBB breakdown in humans is independent of Aβ and tau pathologies [139], but appears to be caused by functional changes of pericytes [137, 139, 140]. However, whether APOE*ε2 also affects BBB integrity in humans and animal models remains elusive.

### Protective effect of APOE*ε2

#### APOE*ε2 and brain structure

Progressive cortical thinning and volume loss occur along the AD trajectory, namely, from cognitively normal to mild cognitive impairment (MCI) to AD [141–146]. However, it remains unclear whether APOE*ε2 reduces AD risk by preserving the cortical structure. Evidence from imaging studies shows no structural difference in cortices between APOE*ε2 carriers and APOE*ε3/3 homozygotes in children and young adolescents [147–149]. However, studies of adults yield conflicting results. Although some investigators report that APOE*ε2 is associated with increased cortical thickness and lower atrophy rate in sub-regions of the temporal lobe relative to APOE*ε3/3 homozygotes in non-demented aged people [150–152], others find no such difference [153, 154]. Nevertheless, APOE*ε2 carriers appear to have better preserved cortical structures than non-carriers among MCI and AD patients [152, 154], a finding that requires validation in larger cohorts.

#### APOE*ε2 and cognition

A plausible explanation of the protective effect of APOE*ε2 against AD may be that APOE*ε2 carriers have better baseline cognition, which sets a higher threshold for cognitive impairment. However, efforts to identify the beneficial effects of APOE*ε2 on cognition in young to middle-aged non-demented individuals have generated mixed results. Although one study reported that non-demented, middle-aged APOE*ε2 carriers perform slightly better in cognitive domains including episodic memory and executive functions [155], *APOE* exerts no effect on intelligence quotient (IQ), memory and school attainment tests in children and college students [156, 157]. Likewise, another study on a community-based cohort in Australia failed to identify APOE*ε2 effects on a battery of cognitive tests in non-demented individuals aged 20 to 60+ [118].

In contrast to observations from young subjects, the cognitive effect of APOE*ε2 in non-demented aged people is more consistent across studies. APOE*ε2 carriers outperform non-carriers in memory tests, visuospatial measures, and global cognition in cross-sectional studies [158–160]. Moreover, longitudinal studies show that APOE*ε2 carriers have lower rate of age-related decline in global cognition [12, 161], episodic memory [119], executive function [120], and verbal learning ability [162]. Interestingly, the protective effect of APOE*ε2 on cognition is more prominent in females than in males [12, 163].

#### APOE*ε2 and longevity

APOE*ε2 has been well-associated with longevity. Cauley and colleagues first reported a higher allele frequency of APOE*ε2 and a lower allele frequency of APOE*ε4 in the elderly than those middle-aged [164]. Although their study focused exclusively on females, similar observations have been reported in French male centenarians [165]. These results have been further validated by cross-sectional case-control studies [166–168] and longitudinal studies [24, 25]. The association between the *APOE* gene locus and longevity has also been confirmed by several case-control genome-wide association studies (GWAS) [19–23].

Despite ample evidence supporting the *APOE* allele-specific effect on longevity, the mechanisms driving the effect remain unknown. Although APOE*ε2 may increase longevity by protecting against AD [169], evidence also suggests a beneficial effect of APOE*ε2 on survival among cognitively normal individuals [24, 25]. Likewise, although dementia is likely the major cause of death among seniors of APOE*ε4 carriers [25], APOE*ε4 also mediates a detrimental effect on survival in non-demented aged people [24]. Furthermore, evidence shows that non-sex-specific cancer reduces life expectancy in APOE*ε4 carriers more than in non-carriers [170].

### APOE*ε2 protects against AD: the clinical evidence

The protective effect of APOE*ε2 against AD was first uncovered in 1994 when the APOE*ε2 allele was found to be underrepresented in AD patients [171, 172]. Compared to APOE*ε3/3 homozygotes, the risk of AD in APOE*ε2 carriers is approximately 50% less [5, 6]. Moreover, AD patients who are APOE*ε2 carriers exhibit slower cognitive decline compared with non-carriers [173]. APOE*ε2 also protects against AD in Down’s syndrome (DS) patients whose amyloid-beta precursor protein (*APP)* gene is triplicated [174]. Amongst DS individuals, APOE*ε2 carriers have reduced risk and delayed age at onset of AD [175–177].

**Fig. 2:**
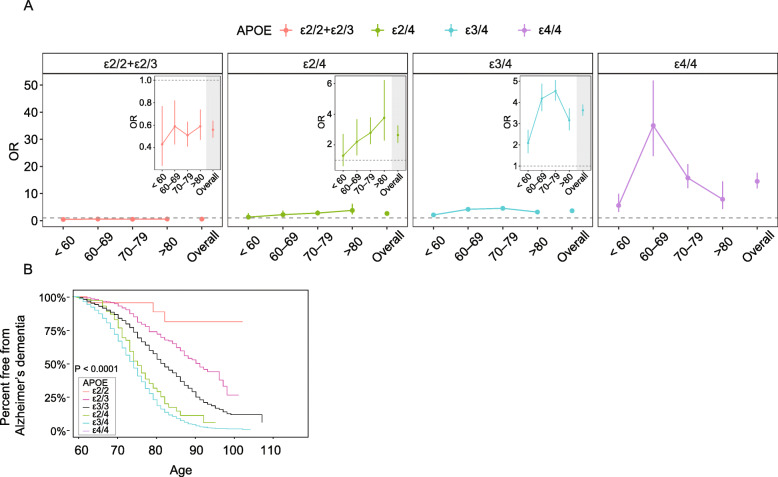
APOE*ε2 protects against AD. **a** Age-stratified odds ratio for AD risk (with APOE*ε3/3 as reference group) in individuals of different *APOE* genotypes. APOE*ε2/2 and APOE*ε2/3 individuals have reduced risk of AD (OR < 1) compared to APOE*ε3/3 individuals and the protective effect sizes are similar in different age groups. In contrast, APOE*ε4 carriers and APOE*ε2/4 individuals, have increased risk of AD (OR > 1) and the effect size varies among different age groups. **b** Kaplan-Meier curves showing the percentage of pathologically confirmed AD cases in individuals of different *APOE* genotypes. APOE*ε2 carriers are less likely to be pathologically diagnosed as AD. The protective effect is more prominent in APOE*ε2/2 homozygotes. **a** A reproduction of published data by Genin, et al., Mol Psychiatry. 2011 Sep;16(9):903-7, with permission. **b** A visual adaptation of a figure from Reiman et al.*,* Nat Commun. 2020 Feb 3;11(1):667, with permission

How demographic factors such as gender, race, and age may modify the protective effect of APOE*ε2 against AD has been investigated. For example, APOE*ε2 appears to be more protective in females than in males [178], but equally protective across ethnicities [5]. Although the effect of APOE*ε4 on AD risk peaks at age 60-69, individuals of different age groups are equally protected by APOE*ε2 [6, 179] (Fig. 2a). Furthermore, APOE*ε2 carriers appear to benefit more from cognitive-enhancing life experiences, such as education and reading, regarding their roles in reducing AD risk than non-carriers [180].

### APOE*ε2 protects against AD: the pathological evidence

#### APOE*ε2 reduces Aβ pathology in humans

The protective effect of APOE*ε2 is more pronounced in pathologically confirmed AD than clinically diagnosed AD [10] (Fig. 2b). Postmortem AD brains from APOE*ε2 carriers have lower densities of Aβ containing neuritic plaques than those from APOE*ε3/3 individuals [7–9], suggesting a slower antemortem Aβ deposition in APOE*ε2 carriers*.* Supporting this, positron emission tomography (PET) imaging in non-demented individuals shows that brain amyloid accumulates at a lower rate in APOE*ε2 carriers than in APOE*ε3/3 homozygotes during aging [11]. Moreover, APOE*ε2 carriers have an older age of amyloid positivity onset than non-carriers [11]. CSF Aβ42 is a widely-used biomarker for AD [181]. Reduced Aβ42 levels in the CSF correlate well with increased Aβ load in the brain shown by amyloid PET imaging [182, 183] or autopsy [184]. Consistent with the imaging study, APOE*ε2 is also associated with higher levels of CSF Aβ42 in middle-aged to aged individuals, irrespective of the cognitive and neurodegeneration status of the subjects [185–187].

APOE*ε2 affects not only the global Aβ load but also the region-specific Aβ deposition. Multimodal neuroimaging in non-demented individuals shows reduced amyloid load in the precuneus in APOE*ε2 carriers compared with APOE*ε3/3 homozygotes [188]. Moreover, the precuneal Aβ burden in APOE*ε2/3 individuals does not increase significantly with age, contrasting to non-carriers [188]. Interestingly, despite ample evidence supporting the protective effect of APOE*ε2 against Aβ deposition, studies show that non-demented APOE*ε2 carriers over 90 years of age (oldest old) have a higher burden of neuritic plaques relative to non-carriers [13, 189], raising the possibility that APOE*ε2 carriers are more resilient to Aβ pathology than non-carriers so that the oldest old individuals can survive better from Aβ toxicity and have cognitive functions preserved. The protective role of APOE*ε2 against Aβ-associated toxicity is discussed in detail below.

#### APOE*ε2 and Aβ aggregation in animal models

How APOE2 affects Aβ deposition has been investigated through crossing 5xFAD mice with APOE-TR mice (denoted as EFAD mice) [190]. One group found that E2FAD mice have similar levels of Aβ42 in the hippocampus at different ages and higher levels of total Aβ42 in the cortex at six months of age compared with E3FAD mice [191]. The lack of protective effect of APOE2 against hippocampal Aβ deposition in animal models has also been shown in a model of PDAPP transgenic mice crossed with APOE-TR mice (denoted as PDAPP/TRE mice) [192]. However, PDAPP/TRE2 animals show lower Aβ load measured by immunohistochemistry in the cortex than PDAPP/TRE3 animals at 18 months of age [192]. The impact of APOE on Aβ pathology has also been investigated through viral-mediated overexpression of human APOE in amyloid mouse models expressing murine *Apoe*. In PDAPP mice, lentiviral-mediated overexpression of APOE*ε2 reduces hippocampal Aβ levels more than APOE*ε3 and APOE*ε4 overexpression [193]. Consistently, Aβ40 and Aβ42 levels in both soluble and insoluble fractions of the brain lysate are reduced with APOE*ε2, but not APOE*ε3 or APOE*ε4 gene delivery in APP/ PS1 mice [194]. Taken together, these studies suggest that the effect of APOE2 on amyloid pathology in animal models can be affected by age, brain region of interest, the strain of amyloid mouse models, and the presence of murine *Apoe*.

#### APOE*ε2 and Aβ production

An imbalance between Aβ production and clearance is considered a crucial event initiating the amyloid cascade in AD [195]. Whether APOE2 impacts Aβ deposition in humans by affecting Aβ production remains inconclusive. Although APOE has a negligible effect on APP processing [196–198], there are conflicting results regarding the role of APOE isoforms in APP transcription. One recent study showed that both lipidated and non-lipidated APOE upregulates *APP* expression in human neurons derived from embryonic stem cells (ESC) or human induced pluripotent stem cells (iPSCs) through the DLK → MKK7 → ERK1/2 signaling pathway. The effect is most prominent for APOE4, followed by APOE3, and then APOE2 [17, 199]. However, the described APOE isoform-specific role in *APP* transcription conflicts with a transcriptomic study showing that APOE2-TR, APOE3-TR, and APOE4-TR mice have similar levels of endogenous murine *App* in the brain (the result can be found through the searchable web interface: https://www.epaad.org/blog/index.php/gene-expresssion-database/) [200].

#### APOE*ε2 and Aβ clearance and degradation

Brain parenchymal Aβ is eliminated through multiple pathways, including cellular uptake, extracellular enzymatic degradation, CSF absorption, clearance via the BBB, and ISF bulk flow [201]. APOE mediates Aβ elimination from the brain in an isoform-dependent manner in which APOE4 mediates Aβ clearance at a lower efficiency than APOE3 [74, 81, 198]. In contrast, APOE2 tends to mediate Aβ clearance across the BBB at a higher efficiency than APOE3 [81, 198]. APOE2 also regulates cellular uptake and degradation of Aβ. One study showed that macrophages in culture from APOE2-TR mice are more efficient in degrading both soluble and insoluble Aβ than macrophages from APOE3-TR and APOE4-TR mice. The higher efficacy of APOE2-associated Aβ degradation is likely related to the enhanced matrix metalloproteinase-9 activity [202]. Additionally, APOE has been shown to mediate soluble Aβ degradation by microglia at an efficacy order of APOE2 > APOE3 > APOE4 [203].

#### APOE*ε2 protects against Aβ toxicity

Previous studies have shown that amyloid load correlates poorly with cognitive impairment and AD severity [204]. Instead, soluble oligomeric Aβ is suggested to be more directly linked to the neurotoxicity in AD brains [204, 205]. The regulatory role of APOE isoforms in Aβ oligomerization has been demonstrated by split-luciferase assays showing that immortalized astrocyte or HEK293 cell-derived APOE promotes Aβ oligomerization with a potency order of APOE4 > APOE3 > APOE2 [206]. However, the in vitro observation of reduced Aβ oligomerization associated with APOE2 was not supported by a study of EFAD mice reporting similar levels of oligomeric Aβ in the soluble fraction of the brain lysate in E2FAD and E3FAD mice [191]. In addition to different modeling systems used, a direct comparison of results from these two studies can be challenging due to the dynamic nature and complex composition of Aβ oligomeric species [205, 207]. Future studies using combinatory approaches (e.g.*,* conformation-specific antibody-based assay or mass spectrometry) to quantify oligomeric Aβ in the brain lysate and CSF of human subjects of different APOE genotypes may help address the question of whether APOE2 reduces oligomeric, toxic Aβ species.

APOE2 also appears to exert anti-toxic effects against Aβ. Both lipidated and non-lipidated APOE2 protect the B12 neuronal cell line against Aβ25-35-induced cell death more than APOE3 and APOE4 [208]. Moreover, hippocampal slices prepared from young adult APOE2-TR mice are more resistant to AD brain lysate or Aβ42-induced LTP suppression than slices prepared from APOE3-TR and APOE4-TR animals of the same age [18, 209]. There is also evidence suggesting that APOE2 expression reduces synaptic loss and neuritic dystrophy in amyloid mouse models [194, 210]. Additionally, APOE2 appears to confer protection for other brain cell types, including cultured pericytes [211] and endothelial cells [212], which potentially constitute indirect pathways for neuronal protection.

#### APOE and Aβ interaction: essential for Aβ deposition?

The essential role of murine APOE in Aβ deposition in animal models has been well-recognized [213]. However, inferring the isoform-specific role of human APOE in Aβ deposition based on studies of murine APOE may be difficult as there is only one APOE isoform in mice, which is structurally and functionally different from human APOE [190, 214]. How human APOE is involved in Aβ deposition is not entirely clear. In vitro studies show that human APOE forms SDS-insoluble complexes with Aβ, irrespective of the lipidation status [215–219]. The complex formation requires the C-terminal lipid-binding domain of APOE [220], and shows APOE isoform-dependency, with lipidated APOE2 binds Aβ at a higher affinity than lipidated APOE3, followed by lipidated APOE4 [219, 221]. Consistently, E2FAD mice have higher levels of SDS-resistant APOE/Aβ complex than E3FAD mice > E4FAD mice in brain lysate [222]. In postmortem human brains, APOE co-deposits with Aβ plaques [216, 223]. Taken together, these studies suggest that APOE-Aβ complex formation can either protect against or promote Aβ deposition, likely in an APOE isoform-specific manner. Interestingly, blocking the interaction between human APOE and Aβ with Aβ12-28P, a synthetic peptide that is homologous to the APOE binding domain of Aβ, reduces brain Aβ levels in APP/PS1 mice crossed with APOE-TR animals [224]. However, Verghese et al. show that APOE has minimal binding with soluble Aβ in human CSF and in the ISF of animal models [225], raising the possibility that Aβ deposition in humans does not require APOE/Aβ complex formation, but instead is affected by a direct seeding effect of APOE on amyloids [226, 227].

#### APOE*ε2 and neurofibrillary tangles (NFTs)

NFTs containing hyperphosphorylated tau represent another pathological hallmark of AD [228–230]. Autopsy studies show reduced NFTs in postmortem AD brains of APOE*ε2 carriers [7–9]. Although the mechanism underlying this reduction is poorly understood, the protective effect of APOE*ε2 against AD tau may be partially mediated through its effect on Aβ deposition, as APOE*ε2 negatively correlates with tau pathology only in Aβ positive but not in Aβ negative individuals [231]. Whether and to what extent APOE*ε2 may protect against tau pathology independently of Aβ in AD remains elusive.

Progress in our understanding of tau pathogenesis in AD is hampered by a lack of sophisticated mouse models that mimic human NFT tau [232, 233]. The widely used tau models, including rTg (tauP301L)4510 mice and Tau P301S/PS19 mice, carry the familial frontotemporal lobar degeneration (FTLD) *MAPT* mutation at the P301 residue, which is not found in AD patients [233]. Thus, results from studies using these models should be interpreted carefully. Bearing this in mind, one study showed that PS19 mice have similar levels of tau pathology and brain atrophy when crossed with APOE2-TR mice versus when crossed with APOE3-TR mice [131]. However, another study found that viral-mediated TauP301L expression induces more tau pathology in APOE2-TR mice than in APOE3-TR mice, suggesting that APOE2 increases the risk of primary tauopathies [30]. Supporting this, APOE*ε2 has been associated with increased risks of PSP and argyrophilic grain disease (AGD) in humans [30, 31]. Future studies to gain mechanistic insights into the impact of APOE isoforms on AD tau require novel animal models that harbor both Aβ and tau pathologies. In addition, the emerging tau PET imaging will permit the exploration of tau pathogenesis in human brains in vivo [234, 235].

### How APOE*ε2 protects against AD: a working model

**Fig. 3:**
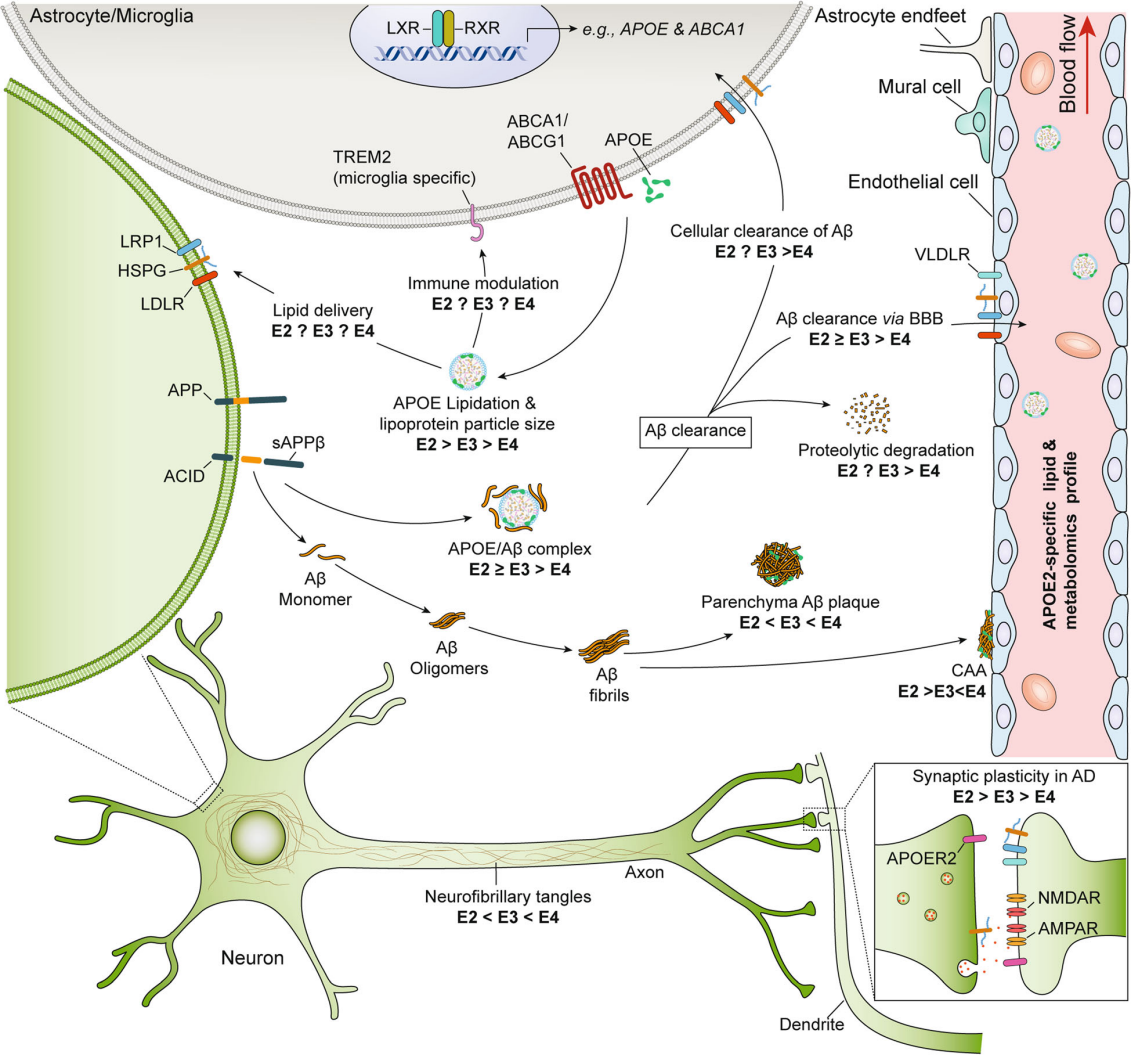
Potential mechanisms underlying APOE2 protective effects against AD. CNS APOE is produced primarily by astrocytes, and also by activated microglia. Newly synthesized APOE is lipidated through cell surface ABCA1 or ABCG1, generating HDL-like lipoprotein particles. In the CNS, APOE2-containing lipoprotein particles are more lipidated than APOE3 and APOE4-containing particles, thus are larger in size. The lipidation of APOE can be modulated by targeting the transcription factors, LXR, and RXR, which regulate the expression of APOE and ABCA1. Lipidated APOE plays a critical role in lipid transport from astrocytes to neurons. Due to hyperlipidation, APOE2-containing lipoprotein particles likely deliver lipids to neurons at a higher efficiency than APOE3 and APOE4. APOE2 may also maintain synaptic plasticity during AD, potentially through interacting with synaptic APOE receptors. During AD pathogenesis, Aβ is produced primarily by neurons through proteolytic processing of APP. APOE regulates Aβ metabolism in an isoform-dependent manner. APOE2 likely mediates Aβ clearance via BBB at a higher efficiency than APOE3 and APOE4. In addition, APOE2 may have a stronger effect in promoting the proteolytic degradation of Aβ by extracellular enzymes. The regulatory roles of APOE in Aβ metabolism may be partially mediated through APOE/Aβ complex formation. APOE*ε2 has also been associated with reduced neurofibrillary tangles in AD patients, though the mechanism is unclear. Additionally, APOE2 may confer protection against AD by affecting the plasma lipid and metabolomics profiles. ACID, intracellular domain of the amyloid-precursor protein; sAPPβ, soluble amyloid precursor protein β

Taken together, APOE*ε2 may protect against AD through multiple, interconnected mechanisms. Based on a growing body of evidence, we propose that hyperlipidation of APOE2 is a central mechanism underlying the protective effect of APOE*ε2 (Fig. 3). Although direct evidence showing increased lipidation of APOE2 relative to APOE3 and APOE4 in human brains is not available, accumulating evidence demonstrates that APOE2 from human CSF [236], immortalized astrocytes [237], as well as primary microglia and astrocyte culture derived from human APOE knock-in mice, are more lipidated than APOE3 and APOE4 [46]. Lipidation substantially impacts APOE binding to receptors and other proteins, such as Aβ [67–69, 238], and also affects APOE catabolism, leading to changes in peripheral and CNS APOE levels [239]. Differential lipidation of APOE isoforms potentially contributes to the distinct cognitive and pathological outcomes in humans of different *APOE* genotypes through both Aβ-independent (e.g.*,* neurotrophic effect, lipid metabolism, synaptic function, and immunomodulation) and Aβ-dependent pathways.

APOE2 may have a greater neurotrophic effect, which maintains neuronal survival and synaptic functions during AD pathogenesis. This is likely achieved by APOE2-mediated lipid metabolism and APOE2-triggered neuroprotective signaling pathways [15–17]. In addition, evidence suggests a critical role of APOE in microglial functions during AD pathogenesis [89, 90, 126–128]. How APOE2 may regulate the immune response of microglia differently than APOE3 and APOE4 remains unclear. Previous studies have shown that promoting cholesterol efflux reduces the immune response of macrophages [240, 241]. Given that APOE2 is a better cholesterol acceptor than APOE3 and APOE4 [242, 243], one may assume a reduced inflammatory response of microglia associated with APOE2 in AD, which requires further investigation. Additionally, hyperlipidation of APOE2 may contribute to reduced Aβ deposition. Supporting this, *Abca1*-knockout increases [244], whereas *Abca1-*overexpression decreases Aβ deposition in PDAPP mouse models [245]. Furthermore, increasing APOE lipidation through pharmacological activation of liver X receptors (LXRs) reduces Aβ deposition in AD transgenic animal models [203, 246, 247]. APOE2 also has been associated with longevity [19–23]. Although the reduced AD risk in APOE*ε2 carriers may contribute to their longer life expectancy, it is also possible that there are unknown anti-aging effects that contribute to their reduced risk of AD through a systemic impact on the whole body. These factors could be APOE2-specific proteins, lipids, and/or metabolites in the plasma [200, 248].

### APOE*ε2 and other proteinopathies

#### APOE*ε2 and TDP-43 proteinopathy

Intracellular TDP-43 inclusion is a shared pathological hallmark of amyotrophic lateral sclerosis (ALS) and FTLD [249]. TDP-43 aggregation is commonly present in hippocampal sclerosis and AD brains [250–253]. Although clinical evidence shows no correlation between *APOE* and ALS risk [254], APOE*ε2/2 ALS patients exhibit decreased glucose metabolism in extra-motor areas compared with APOE*ε3/3 homozygote patients, implying an increased risk of cognitive impairment associated with APOE*ε2 in ALS patients [255]. The impact of APOE*ε2 on FTLD risk remains inconclusive, with APOE*ε2 exerting either no effect or an increased risk of FTLD [256–258].

Pathologically, APOE*ε4 has been associated with exacerbated TDP-43 proteinopathy in FTLD [259]. There is also evidence showing that APOE*ε4 increases the TDP-43 burden in the brain independently of Aβ and tau load, which mediates the increased risk of hippocampal sclerosis in APOE*ε4 carriers [260]. However, the effect of APOE*ε2 on TDP-43 pathology remains unknown.

#### APOE*ε2 and α-synuclein proteinopathy

Dementia with Lewy bodies (DLB) and Parkinson’s disease dementia (PDD) are two neurodegenerative diseases collectively known as Lewy body dementia (LBD) [261]. Pathologically, LBD is characterized by cytoplasmic α-synuclein (αSyn) positive inclusions known as Lewy bodies. α-Syn pathology also affects multiple system atrophy (MSA) [262], and is present in over 50% of the pathologically-confirmed AD brains [263]. Although human studies have shown that APOE*ε4 increases the risk of DLB [264, 265], the impact of APOE*ε2 is less clear. Evidence from a Norwegian cohort suggests a reduced risk of DLB in APOE*ε2 carriers [266], but further validation is required. Although the association between *APOE* and PD has been disproved [267–269], evidence shows an increased risk of PDD in APOE*ε2 carriers [270, 271]. Similar to PD, MSA appears to be also exempted from the impact of *APOE* [272, 273].

Recent studies addressing the effects of APOE isoforms on α-synuclein pathology and related toxicity in vivo have produced interesting findings. αSyn pathology in APOE-TR mice induced by adeno-associated viruses (AAV)-mediated overexpression of human wild type αSyn, or in transgenic mice that overexpress the PD-associated mutant, αSyn (A53T), is exacerbated by APOE4, but not by APOE2 or APOE3 [274, 275]. Although APOE2 protects against αSyn pathology in αSyn (A53T) transgenic mice [274], the protective effect was not observed in the study using the viral-mediated approach [275].

### APOE*ε2 and risks of other neurological disorders

Studies have suggested APOE*ε2 as a risk factor for PTSD, given there is a disproportionately high representation of APOE*ε2 carriers among PTSD patients [28]. Moreover, PTSD patients carrying the APOE*ε2 allele display more severe symptoms [276] and potentially have stronger stress responses than non-carriers [277]. The negative effect of APOE*ε2 on PTSD is also supported by an in vivo animal study showing a slower fear extinction in APOE knock-in mice expressing APOE*ε2 than those expressing other *APOE* alleles [277].

AMD is the leading cause of vision loss in the elderly [278]. The polymorphism of *APOE* has been associated with AMD risk [279–281]. Opposite to the risk profile of AD, APOE*ε2/2 individuals have increased, whereas APOE*ε4 carriers have decreased risk of AMD compared to APOE*ε3/3 homozygotes [29]. In animals, APOE2-TR mice exhibit increased subretinal accumulation of mononuclear phagocytes (MP), retinal degeneration, and choroidal neovascularization than APOE3-TR and APOE4-TR mice at 12 months of age [282]. The detrimental effect of APOE2 in AMD may be partially caused by the APOE*ε2-associated activation of MPs, as blocking the activity of the innate immunity receptor cluster in MPs reduces AMD pathogenies in aged APOE2-TR animals [282].

APOE*ε2 may also modify the risks of other less common neurological disorders. For example, APOE*ε2 has been associated with a reduced risk of Creutzfeldt-Jakob Disease [283], and increased risks of cerebral palsy [284] and Machado-Joseph Disease [285]. However, the evidence should be examined carefully, given the small sample size of these studies.

Large-scale human studies have disputed the association between APOE and multiple sclerosis (MS) [286, 287], whereas the impact of APOE*ε2 on Huntington disease (HD) remains elusive. Despite an earlier report of a younger age at onset of HD in male APOE*ε2/3 patients [288], the observation has not been replicated by others [289].

### APOE*ε2 and cerebrovascular diseases

#### Cerebral amyloid angiopathy (CAA)

CAA is caused by Aβ deposition in cerebral vessel walls [290]. As a common concurrence in AD, CAA mostly affects small arteries and capillaries in the CNS [291]. Despite APOE*ε2 being protective against Aβ deposition in the brain parenchyma, APOE*ε2 carriers are at higher risk and severity of CAA compared to APOE*ε3/3 individuals [26, 27]. APOE*ε2-associated accumulation of Aβ causes amyloid-laden vessels to undergo vasculopathic changes such as fibrinoid necrosis, leading to vessel rupture and resultant hemorrhages in APOE*ε2 CAA patients [292, 293]. In contrast, APOE*ε4 CAA patients more commonly exhibit microbleeds than hemorrhages [27, 294]. APOE*ε2 and APOE*ε4 impact blood vessels of varying sizes, thereby differentially affecting CAA-related pathological outcomes. For example, APOE*ε4, but not APOE*ε2, has been associated with capillary amyloid angiopathy [27]. The mechanism underlying the difference is unclear but possibly related to the differential APOE receptor expression [295, 296], or isoform-specific impact on different vascular cell types [140, 297].

CAA is a common cause of recurrent lobar intracerebral hemorrhage (ICH) [298, 299]. Although ICH-related stroke is relatively uncommon, it is associated with high mortality and morbidity [300]. The APOE*ε2 allele is associated with an increased risk for hematoma expansion in lobar ICH patients, especially in ICH cases with CAA [299], predisposing patients for subsequent hemorrhages. In agreement with that, ICH recurrence within two years of the first event is 18% higher in APOE*ε2 carriers as compared to APOE*ε3/3 individuals [301]. Additionally, the effect of APOE*ε2 on ICH risk appears to be affected by ethnic background, such that APOE*ε2 imposes a higher risk of ICH for Asian than for European individuals [302].

#### Stroke

APOE*ε2, like APOE*ε4, is also a genetic risk factor for stroke [303]. Compared with APOE*ε3/3 individuals, APOE*ε2 carriers are at higher risk for cerebral and cortical infarction [304]. Furthermore, APOE*ε2 is associated with higher chances of both ischemic and hemorrhagic stroke recurrence [298, 301, 302, 305]. Notably, the impact of APOE*ε2 on stroke occurrence may be modulated by age, as the stroke risk in APOE*ε2 carriers decreases significantly after age 70 [304].

### APOE*ε2-inspired therapeutic strategies

As APOE-targeting strategies for AD treatment have been extensively reviewed elsewhere [4, 306–309], herein, we focus on the development of therapies inspired by recent APOE*ε2 studies*.*

#### Viral-mediated APOE*ε2 overexpression

Given APOE2 protects against AD likely due to its greater neuroprotective functions than that of APOE3 and APOE4 (Fig. 3), introducing APOE2 into the brain of AD patients who lack APOE*ε2 may have therapeutic effects. This idea has been tested with amyloid mouse models expressing murine *Apoe*. Viral-mediated overexpression of APOE2, but not APOE3 or APOE4 in the brain at the age when Aβ starts to deposit halts Aβ accumulation and reduces Aβ burden [193, 194], which may be attributed to the increased Aβ clearance in APOE2-expressing animals [194]. Moreover, evidence shows that APOE*ε2 gene delivery into amyloid mouse models with APOE4 expression reduces Aβ levels in the brain [310]. However, since APOE2 increases the risk of certain diseases such as CAA [26, 27], stroke [303], PTSD [28], AMD [29], and primary tauopathy [30], the long-term safety of APOE2 overexpression in human brains should be carefully assessed.

As has been discussed, hyperlipidation of APOE2 lipoprotein may be the central mechanism underlying its protective effect. Thus, pharmacological enhancement of APOE lipidation represents an attractive approach for AD treatment [311–313]. LXRs are transcriptional factors that form heterodimers with retinoid X receptors (RXRs) to regulate the expression of a battery of genes involved in lipid metabolism, including *ABCA1* and *APOE* [314]. Oral administration of the LXR agonists, such as GW3965 and TO901317, increases the protein level and lipidation of brain APOE in mice [203, 246, 247, 315, 316]. Long-term (one month or longer) treatment with GW3965 or TO901317 during early-stage Aβ deposition reduces brain Aβ load and improves cognitive performances of amyloid transgenic animals [203, 246, 247]. However, conflicting reports exist regarding the treatment effect of LXR agonists when there is already substantial Aβ deposition in the brain. One study reported that although TO901317 administration for seven weeks reduces Aβ deposition in the cortex, it yields no impact on the cognition of APP23 mice [315]. Conversely, other studies show that long-term GW3965 or TO901317 treatment rescues cognitive impairments in different amyloid mouse models without affecting the Aβ burden in the brain [317–319].

The potential therapeutic effect of RXR agonists for AD also has been explored, which is best exemplified by the Food and Drug Administration (FDA)-approved drug, Bexarotene. Like LXR agonists, oral administration of Bexarotene upregulates APOE and ABCA1 in mouse brains [320–323]. Studies show that both short-term and long-term treatment of Bexarotene in amyloid mouse models after Aβ has been deposited in the brain restores cognitive performances of the animals, with or without affecting the brain Aβ load [320, 321, 324]. However, the treatment effect of Bexarotene in either cognition or Aβ pathology in animal models has not been replicated by others [323, 325–328]. Interestingly, despite conflicting results from amyloid mouse models expressing murine *Apoe*, there is consistent evidence showing that short-term Bexarotene treatment reverses memory deficit, increases Aβ clearance, and reduces soluble Aβ42 in the hippocampal lysate of amyloid mouse models expressing human APOE isoforms [329, 330].

In humans, Bexarotene treatment increases APOE levels in the CSF [331]. However, the treatment has no impact on brain amyloid load and cognitive functions in AD patients [332]. Moreover, Bexarotene has been reported to cause systemic adverse effects, including hypertriglyceridemia [332], which may limit its potential clinical use in AD patients.

While identifying and testing novel LXR/RXR agonists could be a future direction for AD treatment [333], modulating APOE lipidation by targeting ABCA1 may be a promising alternative option. Overexpression of murine *Abca1* under mouse prion promoter reduces Aβ deposition in PDAPP mice brains [245]. In addition, brain ABCA1 is upregulated by genetic deletion of the small non-coding microRNA (miRNA), miR-33 [334]. Intracerebroventricular infusion of anti-miR-33 oligonucleotides reduces cortical Aβ40 levels in 3-month-old APP/PS1 mice [334]. The activity of ABCA1 may also be enhanced by the APOE mimetic peptide CS-6253 [335]. However, whether CS-6253 induces beneficial effects against AD remains to be tested.

#### Converting APOE*ε4 to APOE*ε2

With the emergence of powerful gene-editing tools such as the CRISPR-Cas system [336–338], generating isogenic iPSC lines from one APOE genotype (normally APOE*ε3/3) to other genotypes becomes efficient and cost-effective [339–341]. Compared to APOE*ε3 cells, isogenic APOE*ε4 cells show dramatic phenotypic changes, including increased Aβ42 and phosphorylated tau in neurons, impaired Aβ uptake and cholesterol metabolism in astrocyte, and reduced phagocytosis of Aβ in microglia [339, 340]. How isogenic APOE*ε2 cells may be functionally different from APOE*ε3 and APOE*ε4 cells remains unclear. Future studies should address the clinical potential of converting APOE*ε4 to APOE*ε2 in vivo as a treatment option for AD.

#### Plasma APOE-based therapy

Although it remains controversial whether and how peripheral APOE may contribute to AD pathogenesis [46, 342], evidence suggests that low plasma APOE levels are associated with increased AD and dementia risk, independent of APOE genotype [343, 344]. Moreover, higher levels of APOE in APOC3-free HDL particles in the plasma have been associated with better cognitive performance and reduced risk of dementia in humans [345]. Since APOE*ε2 carriers have higher levels of plasma APOE [48–51] and HDL [92, 93], whole plasma or plasma APOE-containing lipoprotein particles from APOE*ε2 carriers may hold promise as a therapeutic strategy for AD.

## Conclusions

Despite compelling evidence from human studies supporting the protective effect of APOE*ε2 against AD, the underlying mechanisms remain mostly elusive. APOE*ε2 likely confers protection against AD through both Aβ-dependent and independent mechanisms, both of which appear to be underpinned by increased lipidation of APOE2-containing lipoprotein particles (Fig. 3). To validate the mechanisms proposed in this review, more evidence from humans and animal models is required. Interpretation of data from these studies should be context-dependent, with age, sex, and AD pathology being considered. Furthermore, improved understanding of the roles of APOE2 in other diseases, such as cerebrovascular diseases and different proteinopathies, including tau, TDP-43, and α-Syn pathologies, will aid in the comprehensive assessment of safety regarding APOE2-targeted therapeutics for AD.

In addition to APOE2, other APOE variants have been suggested to protect against AD. For example, the APOE (V236E) variant in the APOE3 backbone has been associated with a significant reduction in AD risk [346]. Additionally, the possession of two copies of the APOE3 Christchurch variant (R136S) markedly delayed cognitive decline in a presenilin 1 (PSEN1) mutation carrier, likely by limiting tau accumulation in the brain [86]. Future studies to validate and to understand the mechanisms underlying the protective effect of these variants will shed light on identifying disease-modifying interventions targeting APOE for AD therapies.

## Data Availability

Not applicable.

## Abbreviations

AAV: Adeno-associated virus
AD: Alzheimer’s disease
AGD: Argyrophilic grain disease
ALS: Amyotrophic lateral sclerosis
AMD: Age-related macular degeneration
AMPAR: α-amino-3-hydroxy-5-methyl-4-isoxazolepropionic acid receptor
APOE: Apolipoprotein E
APOER2: APOE receptor 2
APOE-TR: APOE-targeted replacement
APP: Amyloid-beta precursor protein
BBB: Blood-brain barrier
CAA: Cerebral amyloid
CNS: Central nervous system
DAM: Disease-associated microglia
DLB: Dementia with Lewy bodies
DS: Down’s syndrome
ELISA: Enzyme-linked immunosorbent assay
ESC: Embryonic stem cells
FDA: Food and Drug Administration
FTLD: Frontotemporal lobar degeneration
GWAS: Genome-wide association studies
HD: Huntington disease
HSPG: Heparan sulfate proteoglycan
ICH: Intracerebral hemorrhage
iPSC: Human induced pluripotent stem cell
IQ: Intelligence quotient
ISF: Interstitial fluid
LBD: Lewy body dementia
LDLR: Low-density lipoprotein receptor
LOAD: Late-onset Alzheimer’s disease
LPS: Lipopolysaccharide
LRP1: LDLR-related protein 1
LXR: Liver X receptor
MAP: Mitogen-activated protein
MCI: Mild cognitive impairment
MGnD: Neurodegenerative phenotype
miRNA: MicroRNA
MP: Mononuclear phagocytes
MS: Multiple sclerosis
MSA: Multiple system atrophy
NFT: Neurofibrillary tangles
NMDAR: N-methyl-D-aspartate receptor
PDD: Parkinson’s disease dementia
PET: Positron emission tomography
PSD-95: Postsynaptic density protein 95
PSEN: Presenilin
PSP: Supranuclear palsy
PTSD: Post-traumatic stress disorder
RXR: Retinoid X receptor
TREM2: Triggering receptor expressed on myeloid cells 2
VLDLR: Very low-density lipoprotein receptor
αSyn: α-synuclein
βVLDL: β-migrating VLDL
